# Supplementary material for: Pathophysiological Significance of Neutrophilic Transfer RNA-Derived Small RNAs in Asymptomatic Moyamoya Disease
Source: Cells. 2021 May 1;10(5):1086. doi: 10.3390/cells10051086 (PMC8147334; doi:10.3390/cells10051086)

2D Structure	Local AU	Position

```
2643                        8mer-1a  2674
 5'-aagttTTGTACATCATGTTTTCTAACGGAAAa-3' utr
       | :|| |:|| :  |  : || |||||||
 3'-uuugcACUAU-UGGUG-AUGUGA-UGCCUUUg-5' tRF
                 1615  1413       7 6 5 4 3 2
              3' Pairing           Seed
```

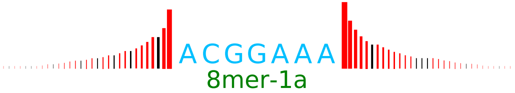

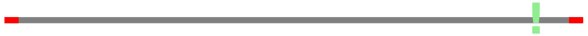

Supplement: Supplementary file 1 [file cells-10-01086-s001.zip › cells-1149161 3rd supplementary materials/Data Supplements/Data Supplement V.zip!/Data supplement 6-mRNAs targets of tsRNAs/tsRNA-Val-AAC-017/Diagrams/tRF-Val-AAC-017_vs_ENST00000612661(MARCKS).pdf]
